# Concrete Durability Performance in Aggressive Salt and Deicing Environments—Case Study of Select Pavement and Bridge Concrete Mixtures

**DOI:** 10.3390/ma18061266

**Published:** 2025-03-13

**Authors:** Olaniyi S. Arowojolu, Milena Rangelov, Somayeh Nassiri, Fouad Bayomy, Ahmed Ibrahim

**Affiliations:** 1 Department of Civil and Environmental Engineering, University of Idaho, Moscow, ID 83844, USA; olaniyi.arowojolu@txdot.gov (O.S.A.); bayomy@uidaho.edu (F.B.); 2 Watershed Technologies, San Francisco, CA 94103, USA; milena@watershedclimate.com; 3 Department of Civil and Environmental Engineering, University of California, Davis, CA 95616, USA; nassiri@ucdavis.edu

**Keywords:** concrete, deicers, salts, pavement, bridge decks, deicing scaling

## Abstract

Transportation infrastructure such as concrete pavements, parapets, barriers, and bridge decks in cold regions are usually exposed to a heavy amount of deicing chemicals during the winter for ice and snow control. Various deicer salts can physically and chemically react with concrete and result in damage and deterioration. Currently, Idaho uses four different types of deicers during the winter: salt brine, mag bud converse, freeze guard plus, and mag chloride. The most often utilized substance is salt brine, which is created by dissolving rock salt at a concentration of 23.3%. Eight concrete mixtures for paving and structural purposes were made and put through a battery of durability tests. Following batching, measurements were made of the unit weight, entrained air, slump, and super air meter (SAM) fresh characteristics. Rapid freeze–thaw (F-T) cycle experiments, deicing scaling tests, and surface electrical resistivity testing were used to test and assess all mixes. Tests with mag bud converse, freeze guard plus mag chloride, and acid-soluble chloride were conducted following an extended period of soaking in salt brine. Two different structural mixtures were suggested as a result of the severe scaling observed in the structural mixtures lacking supplemental cementitious materials (SCMs) and the moderate scaling observed in the other combinations. The correlated values of the SAM number with the spacing factor have been shown that mixture with no SCMs has a spacing factor of 0.24, which is higher than the recommended value of 0.2 and concentrations of acid soluble chloride over the threshold limit were discernible. In addition, the highest weight of calcium hydroxide using the TGA test was observed. For all examined mixes, the residual elastic moduli after 300 cycles varied between 76.0 and 83.3 percent of the initial moduli. Mixture M5 displayed the lowest percentage of initial E (76.0 percent), while mixtures M1 and M2 showed the highest percentage of residual E (83.3 and 80.0 percent, respectively) among the evaluated combinations. There were no significant variations in the percentage of maintained stiffness between the combinations. As a result, it was difficult to identify distinct patterns about how the air content or SAM number affected the mixture’s durability. Class C coal fly ash and silica fume were present in the suggested mixtures, which were assessed using the same testing matrix as the original mixtures. Because of their exceptional durability against large concentrations of chemical deicers, the main findings suggest altering the concrete compositions to incorporate SCMs in a ternary form.

## 1. Introduction

Various deicing chemicals are applied during the winter in cold climates for ice and snow control and to maintain safe road conditions. The low effective temperature, great ice-melting capacity, ease of application, skid resistance, and deicers cost are some of the numerous criteria that go into choosing deicing chemicals [1]. Deicers fall into two categories: inorganic and organic salt. Agricultural salt, calcium chloride, magnesium chloride, and sodium chloride (solid or solution) are all members of the inorganic salt group. Potassium acetate, sodium acetate, calcium magnesium acetate, and potassium formate are all members of the organic salt group [1].

Most inorganic deicing chemicals are chloride-based and cheaper than organic salts and are used more commonly than organic salts [2,3,4]. These salts’ chloride ions have the ability to permeate concrete, and their presence in unreinforced concrete can have both positive and negative effects [2,3,4]. Particularly for concrete that comes into contact with soil. Sulfate attack could be prevented if chloride bonds to the concrete medium (bound chloride). Chloride ions, on the other hand, can cause degradation of the concrete element if they are allowed to freely migrate within the concrete matrix (free chloride) [4]. It is widely known that steel reinforcement’s Fe^2+^ will react with chloride ions to form Fe^3+^ [5]. This conversion is typically linked to increased volume, or corrosion, which would cause the concrete part as a whole to lose mass, strength, and load-carrying capacity [6]. As a result, signs of damage (cracking, spalling) are commonly observed in many roadside barriers, bridge decks, and parapets at the end of the winter season. These damages and deterioration would reduce the service life of the concrete structure and increase the chloride permeability of concrete.

### Mechanisms of Concrete Deterioration

Concrete structures are subject to a mix of chemical and physical attacks from deicing salts. Scaling, salt crystallization, map cracking, and/or cement paste disintegration during wetting/drying cycles and freezing–thawing conditions are typically signs of physical attack. Conversely, the chemical attack typically causes calcium hydroxide (CH) to leak and Oxychloride compounds to develop when CaCl_2_ is applied. This leads to increased permeability, decreased alkalinity, and a loss of concrete’s integrity, strength, and soundness [6].

Given its capacity to lower a solution’s freezing temperature in comparison to other deicers. According to [6], the best deicer for melting snow and ice is magnesium chloride. Even in the absence of freezing–thawing cycles, concrete exposed to magnesium chloride has been known to crack severely due to the development of brucite, Friedel’s salt and magnesium-silicate-hydrate (M-S-H), magnesium oxychloride, and calcium oxychloride [7]. Magnesium oxychloride (M-O-C), calcium silicate-hydrate (C-S-H) and C(OH)2 or CH, M-S-H, and brucite (Mg(OH)_2_) are the main hydration products of Ordinary Portland Cement that are generated when MgCl_2_ reacts with them. Concrete deterioration due to M-S-H development happens progressively [7]. Because salt brine (NaCl) creates osmotic pressures and unexpected phases, it has been shown to exacerbate freeze–thaw damage in concrete [8,9,10].

Investigations on the overall composition, chemical reactions and behavior of MgO-MgCl_2_-H2O compounds in concrete has advanced during the past few decades. Since CaCl_2_ and Mg(OH)_2_ may develop first in the secondary reaction, calcium oxychloride (CAOXY) may be produced. Using a CaCl2 deicer can also result in CAOXY [6]. Because of the internal hydraulic pressure created, CAOXY development in hardened concrete has been described as extremely expansive and damaging [7,11]. At temperatures higher than the water freezing point, calcium oxychloride can develop, although it is unstable at ambient temperature and low relative humidity. Depending on the molar ratios, MOC and CAOXY can also exist in distinct phases, such as CaCl_2_.3Ca(OH)_2_.12H_2_O, CaCl_2_.Ca(OH)_2_. xH_2_O, and CaCl_2_.Ca(OH)_2_); however, depending on the relative humidity and ambient temperature, the phases can coexist and even switch places in Ca(OH)_2_-CaCl_2_-H_2_O [12].

From the foregoing, tri-calcium aluminate (C_3_A) and CH are required for deterioration to occur in concrete exposed to deicing chemicals. Therefore, it is recommended to limit the content of CH in concrete as much as possible [12]. In this direction, supplementary cementitious materials (SCMs) have been used in concrete to reduce the CH content and concrete permeability or to modify the microstructure of concrete [13,14]. A partial replacement of cement with SCMs in concrete has been reported to increase the durability of concrete through pozzolanic reactions that convert CH from the first hydration process to C-S-H, in addition to the dilution effect, which reduces the amount of C(OH)_2_. Ghazy et al. [8] found that the amount of C_3_A in the cement and the amount of CH accessible for chemical reactions in the hydrated cement paste determine how well the CaCl_2_ deicing salt penetrates concrete. Suraneni et al. [13] reported that at 40% cement replacement with fly ash, a low amount of CAOXY (15 g/100 g of paste) would be formed when concrete (w/cm of 0.36) is exposed to NaCl. On the contrary, Suleiman and Nehdi [14] reported that the level of concrete deterioration increased as the amount of fly ash in cement replacement increased when concrete (w/cm of 0.6) was exposed to 5% sodium sulfate.

In the last two decades, research efforts have been intensified to understand the effect of SCMs in reducing salt damage in concrete exposed to deicing chemicals. One of the outcomes of such research has recommended a maximum of 10% silica fume and 25% fly ash as cement replacement in addition to a reduced water-to-cementitious materials (w/cm) ratio [15].

Therefore, the objective of this study was to evaluate current concrete mixtures used by Idaho Transportation Department (ITD) against scaling and freeze–thaw cycling and develop alternative mixtures for the aggressive deicing and chloride environment [16]. The results of this study would assist highway agencies and practitioners in understanding the physical and chemical deterioration in concrete exposed to deicing chemicals, as well as the effectiveness of the application of SCMs as mitigation measures to inform the mixture design, construction practice, and code specifications.

## 2. Materials, Specimen Preparation, and Curing

Slab, prismatic, and cylindrical specimens were cast for the tests in this investigation in accordance with ASTM C192 [17]. The cylinder specimens were used for compressive strength (ASTM C39) [18], wetting-and-drying cycles [19], surface electrical resistivity (ASTM C1876) [20], and continuous soaking tests [21]; the prisms were used for the freezing and thawing test (ASTM C666) [22], and the slabs were used for resistance to deicing scaling (ASTM C1543) [23]. The concrete cylinders were 100 × 200 mm, the prisms were 75 × 75 × 275 mm, and the slabs were 285 × 285 × 85 mm in size as specified by ASTM standards for each test.

The concrete mixtures were divided into two categories: the first category replicates the actual concrete mixes used by ITD for bridge and paving applications, while the second category consists of alternative concrete mixtures as mitigation measures for the first mixture category. In the first category, the concrete aggregates were locally sourced, and the binder content, type, and water-to-cement (w/cm) ratio replicate field mixture design for bridge construction and highway paving. The second category was similar to the first category except for the binder content that was changed from binary to ternary, as summarized in Table 1a. In the ternary mixtures, cement was replaced with a 10% silica fume and 20% coal fly ash. Table 1b shows all the coarse and fine aggregates used in all mixtures.

The physical and chemical properties of the Ordinary Portland Cement (OPC), silica fume, and fly ash are summarized in Table 2. As shown in Table 2, Fly Ash was categorized as a class-F Fly Ash. Fresh concrete as described in ASTM C143 [24], ASTM C231 [25], and ASTM C138 [26], respectively, were performed on every concrete batch. Freshly created specimens were wrapped with plastic covers to stop evaporation. After casting, the molds were taken out 24 ± 8 h later. Following demolding, the samples were moist cured in a fog chamber at 21 ± 1 °C with a relative humidity (RH) of greater than 95 percent, as specified by ASTM C511 [27] before exposure to a chloride environment.

A super air meter (SAM) H2784 was used according to ASTM C231 [25]. Using this technique made it possible to obtain a SAM number that could be connected with the typical distance between the concrete mixture’s air voids. A large void spacing means concrete is highly susceptible to deicing scaling and freeze–thaw deterioration. According to ACI 201 [28], a maximum spacing factor of 0.2 mm was recommended for concrete exposed to deicing chemicals and freeze–thaw cycles.

## 3. Test Methods

### 3.1. Environmental Exposure (Conditioning) for Deicing Scaling Evaluation

**Figure 1:**
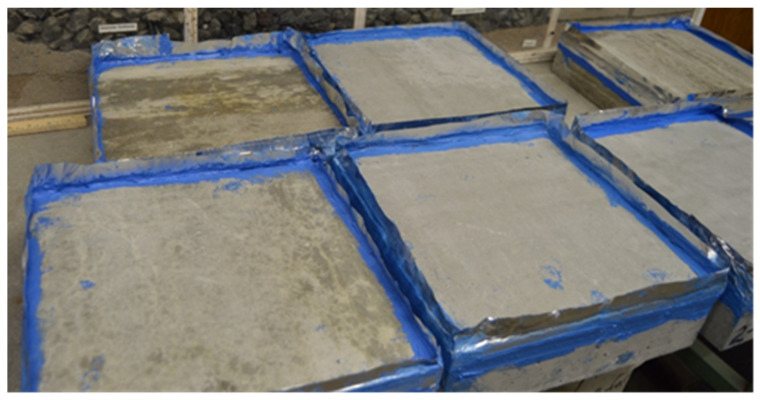
Slab specimen used for deicing scaling resistance test (Source: [16]).

When the top surface of the slab was ponded with deicing chemicals at 28 days of age, as seen in Figure 1, the four upper corners of the 285 × 285 × 85 mm slabs were covered with aluminum tape and sealed with silicone sealers to stop leaks. The slabs’ upper surfaces were coated with 6.35 mm of deicing agent and stored for 16 to 18 h at a freezing temperature of −18 ± 3 °C. The frozen specimens were removed from the freezing chamber and maintained at the room temperature range of 23–25 °C and relative humidity of 45 to 55% for 6 to 8 h according to ASTM C 672/C 672M [29]. Every five cycles, the chosen deicing agent was supplied to ensure enough solution depth and thorough surface flushing. The deicing chemicals (summarized in Table 3) were selected based on the type and concentration used in Idaho state regions where deteriorated concrete structures were identified. The application of a 23.3% concentration of salt brine on mixtures M1 and M6 is due to the high amount of snow annually received in Idaho’s northern region. Following 5 to 50 cycles, the specimens were visually examined and given a surface condition rating between 0 and 5, where 0 denotes no scaling and 5 denotes significant scaling, as shown in Table 3.

Similarly, the cylinder specimens were continuously soaked in deicing chemicals for 90 days to evaluate the deterioration of the concrete microstructure and its effect on compressive strength. In accordance with ASTM C39, the specimens were tested under the standard compression test after 90 days to examine compressive strength [18]. On the same day that the cylinders immersed in deicing agents were evaluated, three control cylinders that had been moist cured in the humidity chamber for ninety days were tested under compression. The strength decrease following soaking was correlated using the reference specimens.

### 3.2. Acid Soluble Chloride Test

The slab specimens used for the deicing scaling test were cored at the center at a 12.5 mm depth interval using a 25 mm coring bit to obtain samples for the acid-soluble chloride test, ASTM C 1152 [30]. This test was performed to determine the chloride concentration profile along the depth of the slab. The cored samples were then made into a solution by dissolving the cored sample powder in acid and titrated against 0.05 N Silver Nitrate (AgNO_3_).

### 3.3. Calcium Hydroxide and Oxychloride Contents

Thermogravimetric analysis was conducted on the ground hydrated cementitious pastes exposed to the deicing chemicals after continuous soaking for 90 days to determine the CH content of the paste using the mass loss between 380 and 460 °C. The thermal degradation and stability of the mixtures were conducted on a 4–5 mg sample using a Perkin–Elmer TGA-7 instrument (Shelton, CT, USA) in a temperature range from 30 °C to 900 °C at a rate of 10 °C/min. The data from the TGA were analyzed using the Pyris v13.3 software. Low-temperature differential scanning calorimetry (LT-DSC) was used to evaluate the formation of oxychloride compounds, as suggested in [31].

### 3.4. Rapid Freeze–Thaw Cycling Test

**Figure 2:**
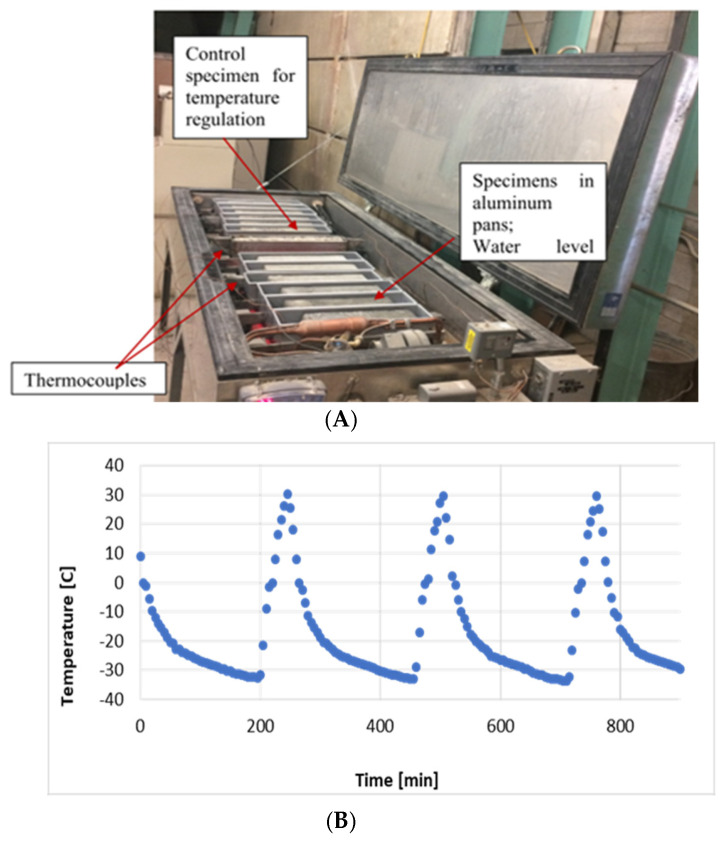
(**A**) Freeze–Thaw (F-T) Cabinet with Concrete Prisms Subjected to F-T testing. (**B**) Temperature exposure cycles [16].

In accordance with ASTM C666, the freeze–thaw (F-T) resistance test was carried out [22]. As previously mentioned, the prism specimens were unmolded and allowed to cure for 14 days in the moist curing room. The prisms were then set up in the F-T cabinet after being placed in aluminum pans (Figure 2). To create a 3 mm water level surrounding each specimen, water was put into each pan from the bottom. During the 300 F-T cycles of testing, the water level remained constant. The exposure temperature is alternated between 4 and −18 °C and between −18 and 4 °C for intervals of two to five hours in the standard F-T cycle. As shown in Figure 2, the F-T system automatically regulates the temperature in the F-T cabinet using a control specimen. Four thermocouples (TCs) that were affixed to the specimens and used for temperature monitoring during the testing confirmed that the cabinet was operating properly.

### 3.5. Surface Resistivity Test

The surface electrical resistivity of cylindrical specimens was measured using a Wenner four-probe, equally spaced, co-linear electrodes resistivity meter according to the procedure in ASTM C1876/AASHTO T358 [20]. The test was conducted on three samples for the same concrete mixtures. Each specimen was labeled at four quadrants to ensure consistency, which stand for 0°, 90°, 180°, and 270°. Two readings were taken in each quadrant in the sequence of the marked angles. The average of the three resistivity measurements was then used to determine the specimen resistivity. Lastly, standard compression in accordance with ASTM C39, the compressive strength test was performed on three soaked and un-soaked specimens per mixture [18]. ACI 209.2R-08 was used to forecast the reference specimens’ compressive strength for ages longer than 28 days [32]. This is provided by the following equation:

(1) $$f_{cmt}=\left[\frac{t}{a+bt}\right]f_{cm28}$$

where

$f_{cmt}$ is the compressive strength at time t, in days, and $f_{cm28}$ is the 28-day compressive strength [32].

*a*, *b* are constants depending on the type of cement used and method of curing, and *t* is the age of the concrete in days.

### 3.6. Scanning Electron Microscope (SEM)

Remains of the slabs used to prevent deicing scaling were inspected under a scanning electron microscope at different depths to determine the depth of penetration of the deicing chemicals. Chemical compounds were shown to develop, but additional testing, such as thermal gravimetric measurement, would be necessary to precisely identify the compound form.

## 4. Results and Discussion for Original Mixtures

### 4.1. Deicing Scaling Resistance

**Figure 3:**
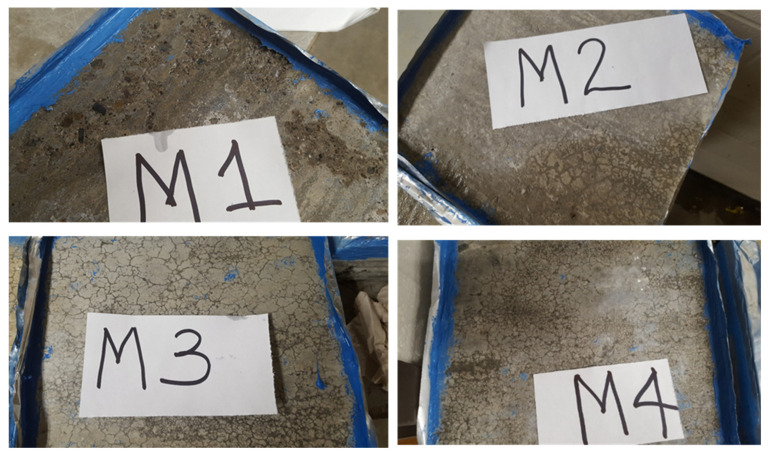

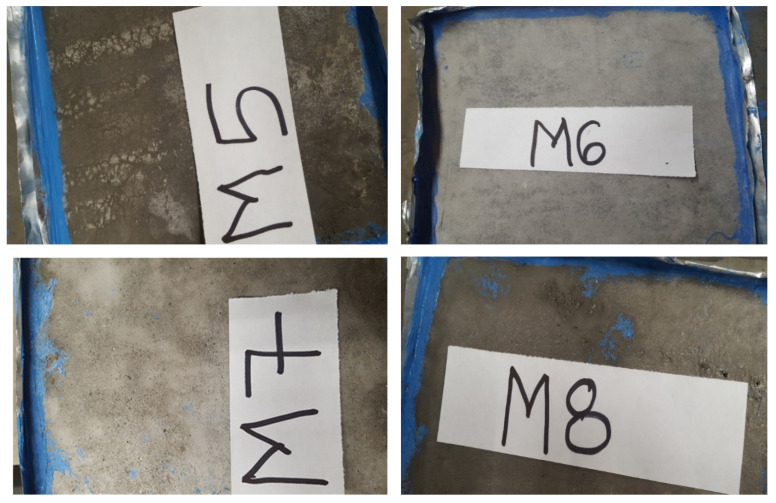
Deicing Scaling Damage of Concrete Slabs (M1–M4) exposed to deicing chemicals ponds on the top of the specimens [16].

After the first 20 cycles of exposure, signs of moderate scaling began to manifest on the slab from mixture M1 (Figure 3), while no sign of deterioration was observed on other mixtures (M2, M3, M4, and M5). At the end of the 50 cycles, severe scaling was observed on the M1 slabs, while M2 slabs displayed minor scaling, and M3 and M4 displayed ASR crack pattern and moderate scaling, as shown in Figure 3. The severe scaling observed on the M1 slab after the first 20 cycles could be the result where cement pores became more saturated after the concrete surface was damaged, and the repeated cyclic of freezing and thawing damaged the finer capillary of the concrete pore structure leading to higher cracking, which allowed deeper salt penetration into the concrete. In one of the field locations, where mixture M1 was used, a sample was taken by coring, and the sample was compared with the laboratory result. The close agreement of the field sample with the laboratory result validates the laboratory testing because the field sample also displayed a severe scaling, with the coarse aggregate exposed. As summarized in Table 1a, the M1 mixture does not have any SCMs but has an adequate water-to-cement (w/cm) ratio and entrained air content as recommended by ACI 318 [15].

The correlated values of the SAM number with the spacing factor are shown in Table 1a. It could be observed that M1 has a spacing factor of 0.24, which is higher than the recommended value of 0.2. The severe scaling of M1 could be attributed to the absence of SCMs and the high spacing factor. The addition of SCMs to concrete mixtures M2 and M5 improved the deicing scaling resistance of the concrete, where minor and moderate scaling were observed, respectively. Based on these observations, it is sufficient to say that the spacing factor is not the only requirement for concrete resistance to deicing scaling. Mixtures M3 and M4 contained SCMs but displayed moderate scaling in addition to alkali–silica reaction (ASR). As shown in Table 1a, the spacing factor of the concrete mixtures was higher than the recommended values of SAM. Some researchers [33,34] have reported that deicing chemicals (salt brine and Mag Bud Converse) can supply more alkalis, which react with reactive aggregate and accelerate ASR formation. There is a high possibility that the aggregate used for M3 and M4 was reactive [34]; hence, ASR damage was observed. MgCl_2_ lowers the pH value of concrete due to the formation of brucite [35,36]. To confirm the ASR observed in M3 and M4, TGA is discussed later in the following sections.

### 4.2. Acid Soluble Chloride Test Results

Because of the significance of those mixes in reinforced concrete elements, this test was conducted on mixtures (M1, M4, and M5) as well as additional mixtures (Table 4). The likelihood of corrosion increases if the concentration of chloride at the rebar level surpasses the ACI 222 [37] threshold value. The chloride concentration that has permeated through the concrete after being exposed to the deicing agent shows that the M1 concrete mixture had the best chloride transport characteristics, as shown in Table 4. At a depth of up to 25 mm, concentrations over the threshold limit were discernible. Recall that in the scaling resistance test, the identical mixture showed severe scaling. The M5 mixture showed a similar outcome. It should be noted that Shi et al. [38] showed that salt brine has more penetrability into concrete than Mag bud converse, and it diffuses into the concrete more quickly.

### 4.3. TGA and LT-DSC Test Results

The (CH) amounts per 100 g paste for the mixtures are summarized in Table 5. A higher amount of CH was obtained in mixture M1, which was as expected because the mixture did not contain SCMs that would have consumed the CH during the secondary hydration reaction. However, the CH produced was higher in mixtures with a higher w/cm ratio. This is because hydration would be impeded at a lower w/cm, as insufficient water would lead to incomplete hydration.

In the mixtures exposed to salt brine, sodium–aluminosilicate–hydrate (N-A-S-H) was the main reaction product, while M-O-C was the main reaction product when the mixture was exposed to Mag-bud converse. Although the amount of MOC and N-A-S-H per 100 g of paste required to cause damage has not been identified in the literature, Suraneni et al. [13] recommended keeping the amount of CAOXY below 15 g/100 to reduce damage caused by CAOXY. However, the exact amount is quite complex to determine because the damage in concrete exposed to the deicing chemical is a function of many factors.

### 4.4. Rapid F-T Cycle Test Results

Once all specimens thawed completely after 300 cycles, they were examined for mass loss and modulus of elasticity (E) using a Metriguard 239 stress wave timer and a non-destructive (ND) sonic pulse velocity characterization.

Table 6 shows for all examined mixes, the residual elastic moduli after 300 cycles varied between 76.0 and 83.3 percent of the initial moduli. All investigated mixes demonstrated satisfactory F-T resistance when taking into account the requirements of [22], which set a failure limit of 60% of initial E. Mixture M5 displayed the lowest percentage of initial E (76.0 percent), while mixtures M1 and M2 showed the highest percentage of residual E (83.3 and 80.0 percent, respectively) among the evaluated combinations. There were no significant variations in the percentage of maintained stiffness between the combinations. As a result, it was impossible to identify distinct patterns about how the air content or SAM number affected the mixture’s durability. As indicated in Table 1a, M3 and M4 achieved the SAM number of 0.2 mm, which was the value advised by ACI 201 [28,39]. Nevertheless, these two mixes showed sufficient resistance to F-T.

**Figure 4:**
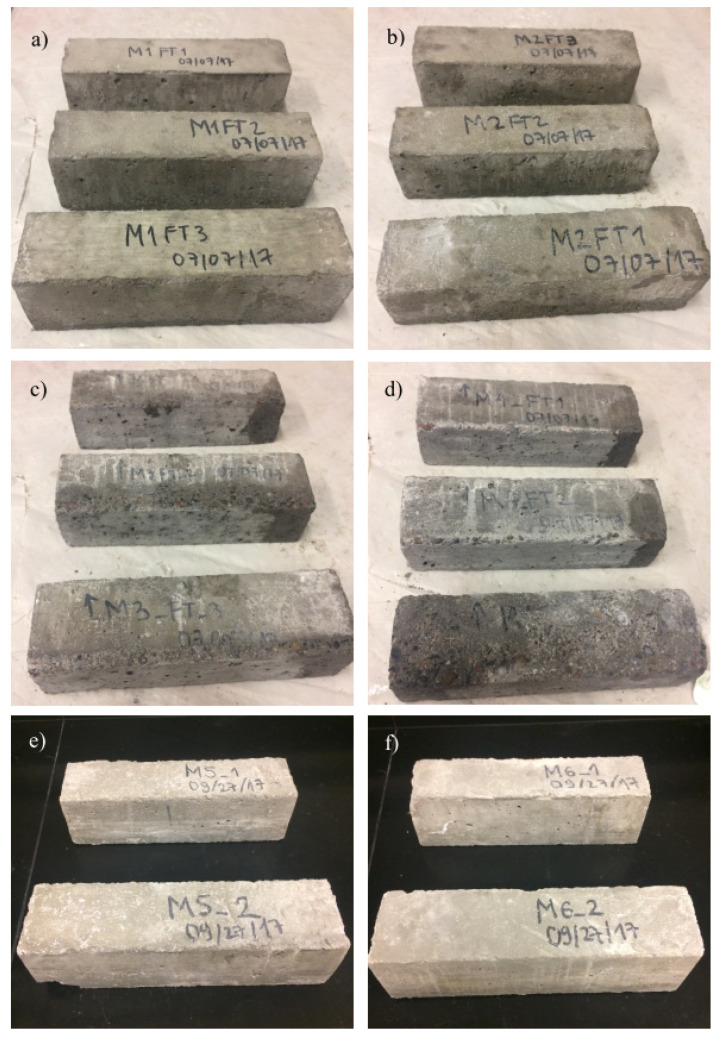

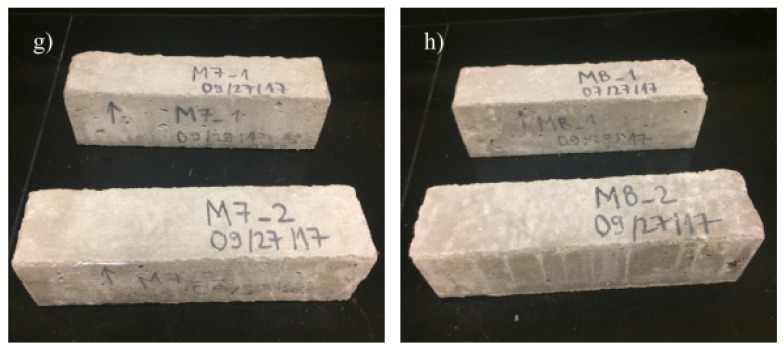
Freeze and Thaw Results for all mixes (**a**) M1, (**b**) M2, (**c**) M3, (**d**) M4, (**e**) M5, (**f**) M6, (**g**) M7, (**h**) M8 [16].

The studied specimens showed mass losses of less than 0.6 percent as a result of freezing and thawing, as shown in Table 6. The two structural mixes from M3 and M4 were the two mixtures with the greatest mass losses. Figure 4 shows severe surface scaling at the bottom of the tested prims, which contributes to the mixtures’ comparatively substantial mass losses. The fact that specimens cast from M3 and M4 kept 79.7 and 76.9 percent of the original E suggests that these specimens’ integrity is not significantly weakened by the influence of F-T cycles. However, the serviceability of structures cast out of these combinations might be hampered by surface scaling, especially when deicing agents are used. While there is no clear association between mass loss and air content, a comparison with test results for fresh concrete (Table 1a) indicates that mixtures with higher SAM values also showed higher mass loss. When M3 and M4 are taken out of the equation, Figure 4 demonstrates that the tested specimens had some calcium leaching on the surface from constant contact with water, some surface damage, and generally good structural integrity that was consistent with the above-mentioned satisfactory F-T performance.

### 4.5. Surface Resistivity Results

Research shows the surface resistivity of concrete is an indicator of the ease at which chloride would diffuse through the hardened concrete [20]. The risk of chloride diffusion through the concrete would increase the potential of steel corrosion in reinforced concrete members [37]. The average resistivity values at 28 days of the concrete mixtures are shown in Table 7. It could be observed that mixture M1 has the lowest resistivity value. The resistivity value correlates well with the concentration of acid-soluble chloride, as presented in Table 7. Mixture M1, with the lowest resistivity value, showed the highest amount of soluble chloride.

### 4.6. Continuous Soaking

**Figure 5:**
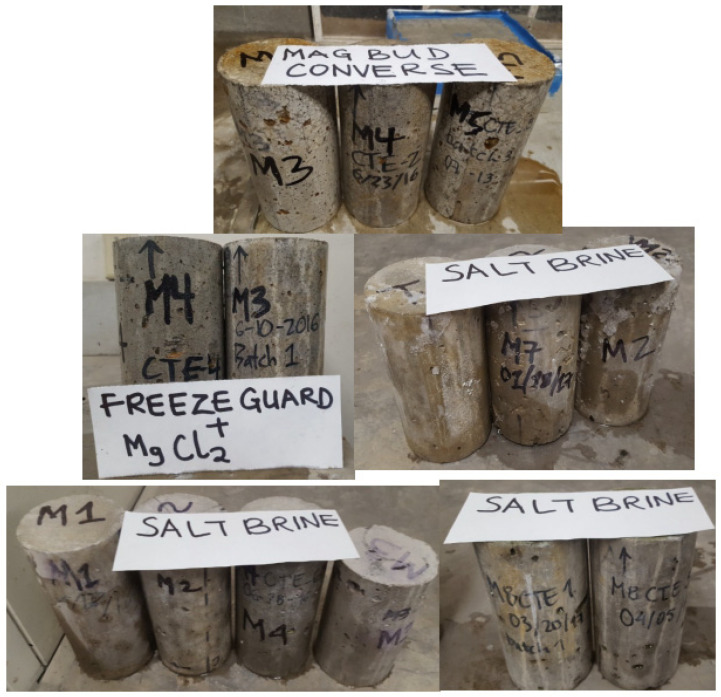
Concrete cylinders after soaking in deicing salt for ninety days straight [16].

In order to assess the impact of deicing agents on the compressive strength of concrete, the cylinder specimens were submerged in them for ninety days, as previously mentioned. While the specimens soaked in salt brine showed mild to moderate deterioration, with the exception of M2, which showed major concrete spalling, the specimens soaked in freeze guard (Figure 5) and magnesium chloride (M3 and M4) showed heavy deterioration of alkali silica reaction accompanied by spalling and wide cracks extended to the entire surface of the slabs. Table 8 provides a summary of the outcomes obtained. It is evident that M1 and M3 mixtures had modest loss of strength, whereas M4 and M5 mixtures saw the greatest loss of strength. According to results, the presence of M-O-C in concrete decreases the C-S-H gel, increases the void within the concrete, and, thus, lowers the concrete’s compressive strength [13]. Similarly to this, concrete exposed to salt brine would form N-A-S-H instead of C-S-H, which would reduce the quantity of C-S-H that could bind the aggregate together and result in a loss of compressive strength [13,40]. The specimens are displayed in Figure 5 following the prolonged soaking.

The cylinders utilized for continuous soaking were subjected to a compressive strength test. The specimens underwent standard compression test (ASTM C39) after being continually bathed in deicing agents for ninety days. Table 8 provides a summary of the outcomes obtained. It is evident that the M3 mixture experienced very little strength loss, but the M5 and M6 mixtures experienced the most strength loss. Following a 90-day soak period, at least two specimens were subjected to axial compression testing. Two unsoaked control cylinders that were kept cured until the testing day were used to compare the results.

### 4.7. Scanning Electron Microscopy (SEM) and Energy Dispersive X-Ray Analysis (EDX)

**Figure 6:**
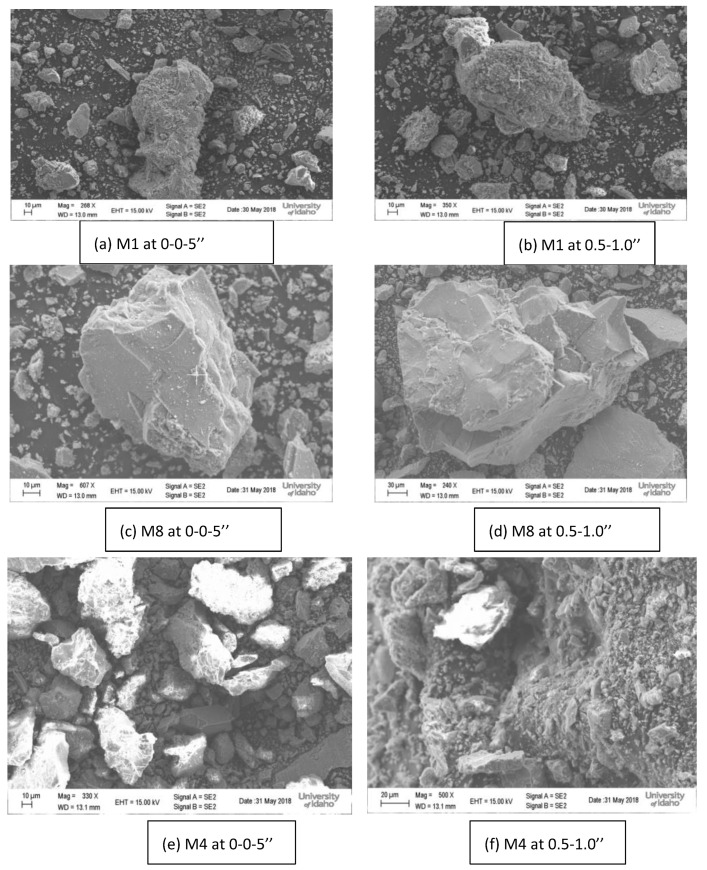
Scanning Electron Microscopy Images for selected mixtures [16].

**Figure 7:**
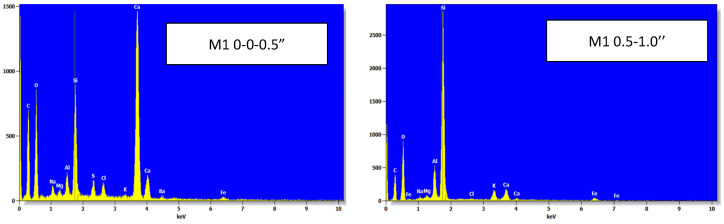
EDX Images for mixture (M1) [16].

In order to determine the possibility of the production of chemical compounds in the concrete microstructure and at various depths. Samples of concrete powders were used for deicing resistance and were taken out of the slabs and inspected using scanning electron microscopy. The energy dispersive X-ray test (EDX) was performed on the concrete samples in order to assess the chemical analysis of the concrete’s constituents. At a depth of one inch, the concrete samples exposed to salt brine showed a significant chloride concentration. Although the SEM pictures (Figure 6) show the presence of new chemical compounds in the concrete, a thorough chemical investigation or the thermal gravimetric test was required to identify the substance. The EDX in Figure 7 shows the presence of chloride in the mixture at a specific depth, which is particularly noteworthy. Since the chloride concentration could not be ascertained, the chloride concentration would be ascertained using acid soluble chloride and compared to the values recommended by the American Concrete Institute (ACI 222).

### 4.8. Alternative Concrete Mixtures

After reviewing the results from the original mixtures, alternative ternary blend concrete mixtures were proposed, batched, cured, and tested, as shown in Table 1b. The alternative mixtures investigated were the structural mixtures (M1, M4, and M8). The proposed mixtures ingredients were based on the reported literature and were developed by replacing OPC with 20% Fly Ash and 10% Silica fume by weight, as summarized in Table 9. The composition of the alternative cementitious materials mixtures was based on data found in the literature but with modifications. A Binary and ternary combination of cementitious materials was proposed, but only the results of the ternary mixtures are presented in this study.

The 40 percent fly ash replicates showed the greatest slump among the three mixture groups. Slump was, therefore, not measured for the fly ash replicate for M1 and M4. Furthermore, compared to the other M4 samples, the air content of the M4_40FA combination was much lower (2 percent for M4_40FA against 4.3 to 5.6 percent for other M4 duplicates).

## 5. Results and Discussions for Alternative Mixtures

### 5.1. SAM Number

The SAM number for mixtures (M1, M4, and M8) has shown values between 0.25 and 0.54. The trend of the effect of SCMs on SAM numbers could not be clearly recognized. However, the SAM numbers were well above the values of the original concrete mixtures, as shown in Table 9. The spacing factors are well below the maximum recommended value of 0.2 mm.

### 5.2. Scaling Resistance

**Figure 8:**
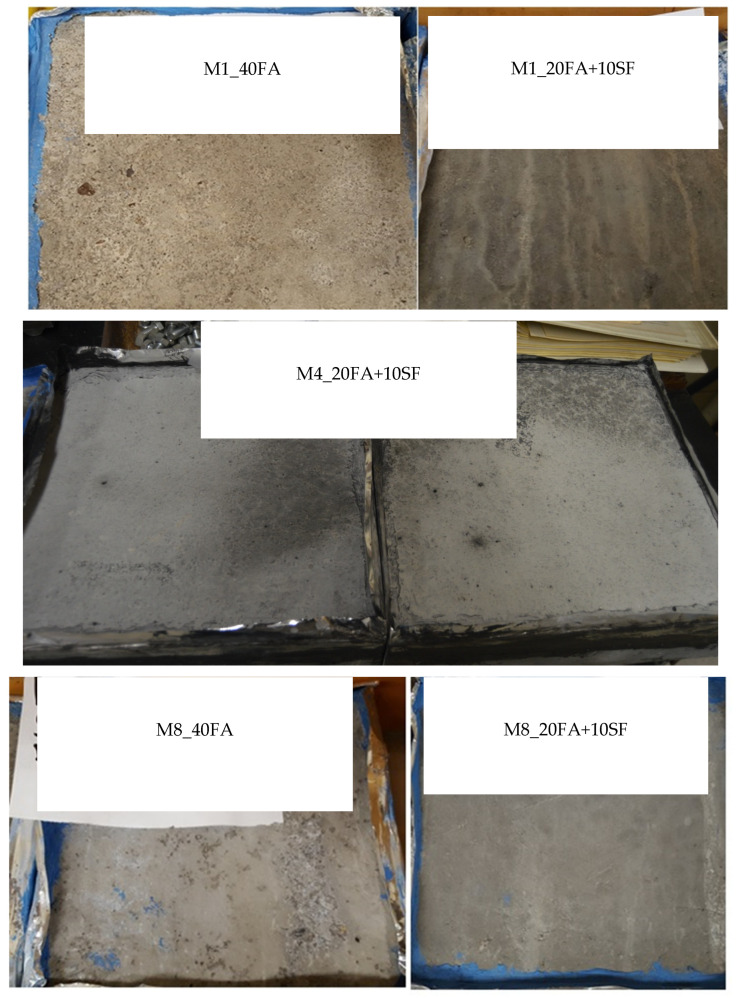
Specimens Tested for Deicing Scaling for M1, M4 and M8 [16].

The mixtures were tested for deicing scaling resistance, while the visual rating is summarized in Table 9. As shown in Figure 8, none of the specimens displayed visible scaling, and this could be a result of the addition of SCMs, such as the high entrained air content, and the presence of silica fume, which could ultimately reduce the amount of CH in the concrete. Reducing the CH content would inhibit the reaction of CH with deicing chemicals. The reduction in the CH and oxychloride content was confirmed by the TGA and LT-DSC tests, respectively, as summarized in Table 9.

### 5.3. Rapid F-T Cycle Test

Before E testing, the mass loss was measured at the end of each 30-cycle. After 300 cycles, the mass losses of every tested mixture were less than 1.0 percent. The M1 replicate, which included 40% fly ash, had the greatest mass loss. After 300 F-T cycles, the same combination likewise held onto about 90% of E. The mass loss of the mix M4_40 was relatively minimal (less than 0.2 percent after 150 cycles), despite not meeting the 60 percent E criterion. According to these findings, mass loss by itself might not be a reliable indicator of mechanical property deterioration on the F-T test.

**Figure 9:**
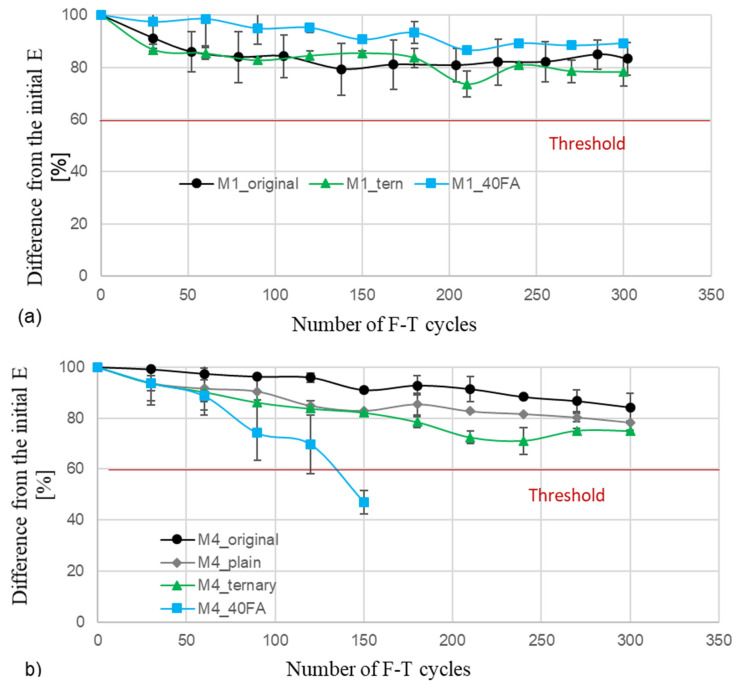

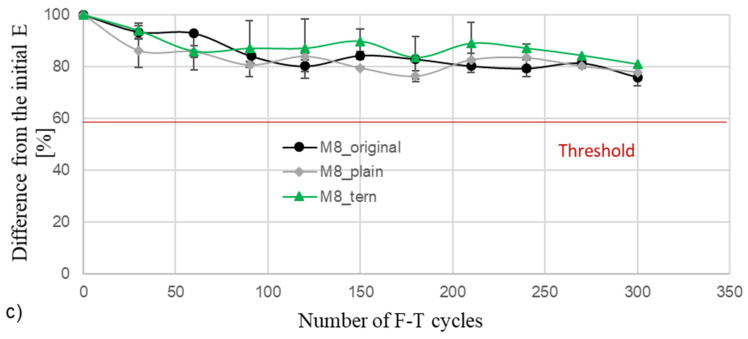
Elastic Modulus variations in F-T Tests for the structural mixtures (**a**) M1, (**b**) M4, and (**c**) M8 [16].

Following the 300-cycle marker specified in ASTM C666, the residual E of all tested mixes is greater than 60% of the initial E. The replication of mixture M4 with 40% fly ash is the lone exception; after 150 cycles, there was a decline in E of more than 60%. After 300 cycles, M1 replicates retain 78–89% of their initial E, with mixture M1_40FA showing the highest residual E. Regarding M4, the replicates that fulfilled the requirements kept between 74 and 84% of their E value. Mixture M4_original had the greatest residual E in its group. After 300 cycles, M8 replicates kept 76–81 percent of their E, with the ternary mixture performing the best, as demonstrated in Figure 9.

### 5.4. Surface Resistivity and Acid Soluble Chloride Test

As shown in Table 10, the surface resistivity test was conducted on the new alternative combinations in the same manner as the original mix. It is evident that all of the mixes displayed very low levels of corrosion risk due to chloride penetrability. The low chloride content values along the slab’s depth are in good agreement with the concrete’s high resistivity values. Note that, with the exception of M1 mixes, the main distinction between the ternary mixtures and the majority of the original mixtures is the addition of 10% silica fume. Every chloride concentration value is below the threshold levels needed to start corrosion.

## 6. Conclusions

In this study, the performance of paving and structural concrete mixtures exposed to aggressive salt and deicing environments was evaluated. Alternative concrete mixtures with more SCMs were developed and tested under the same aggressive environment. The structural mixture (M1) showed severe scaling, according to the test results, whilst the other specimens displayed mild to moderate scaling.

The correlated values of the SAM number with the spacing factor have shown that M1 (no SCMs) has a spacing factor of 0.24, which is higher than the recommended value of 0.2. It is sufficient to say that the spacing factor is not the only requirement for concrete resistance to deicing scaling.

M1 concrete mixture had the best chloride transport characteristics. At a depth of up to 25 mm (rebar location), concentrations of acid soluble chloride over the threshold limit were discernible. Other mixtures performed well in terms of chloride diffusion that was lower than the ACI 222 limit.

The highest weight of calcium hydroxide using the TGA test was observed in mixture M1, which was as expected because the mixture did not contain SCMs. However, the CH produced was higher in mixtures with a higher w/cm ratio. This is because hydration would be impeded at a lower w/cm, as insufficient water would lead to incomplete hydration.

Mixtures with no SCMs, which prevent the formation of calcium oxychloride (CAOXY), may be the cause of the severe scaling. When M2 (paving) and M3 (structural) mixtures were exposed to magnesium chloride under multiple cycles of deicing chemicals, they displayed some evidence of an alkali–silica reaction (ASR) due to the reactive aggregate used in those mixtures.

Following 300 F-T cycles, a relatively high percentage of residual elastic modulus and relatively moderate mass losses show that the investigated mixes operate satisfactorily under the rapid freeze–thaw (F-T) cyclic test. Each of the studied mixes had residual elastic moduli after 300 cycles that varied from 76.0 to 83.3 percent of the original moduli. Among the combinations that were analyzed, mixture M5 had the lowest percentage of initial E (76.0 percent), whereas mixtures M1 and M2 had the highest percentage of residual E (83.3 and 80.0 percent, respectively). There were no significant variations in the percentage of maintained stiffness between the combinations. As a result, it was difficult to identify distinct patterns about how the air content or SAM number affected the mixture’s durability. After 300 cycles, the paving mixture M5, which contained 20% fly ash, showed the least amount of mass loss (1.61 percent). Although all of the mixtures containing 40% fly ash functioned effectively during the F-T cycles, with the exception of M4-40, they demonstrated adequate resistance against deicing scaling.

In addition, the average resistivity values at 28 days of the concrete mixtures have been recorded. It could be observed that mixture M1 has the lowest resistivity value. The resistivity value correlates well with the concentration of acid-soluble chloride. Mixture M1, with the lowest resistivity value, showed the highest amount of soluble chloride. In every durability test, the ternary mixtures—which comprise fly ash and silica fume—performed quite well.

The results of the M1 and M8 structural mixtures showed that, except for ternary mixtures including fly ash and silica fume, concrete samples were significantly damaged by salt brine deicing chemicals produced at a concentration of 23.3%.

## Figures and Tables

**Table 1:** (**a**) The proportion of tested concrete mixtures and their tested entrained air, SAM number, and spacing factor. (**b**) The proportion coarse and fine aggregates of tested concrete mixtures.

**(a)**									
**Mixture No**	**Mixture Application**	**Binder Type**	**Cement Content: kg/m^3^**				**Entrained Air**	SAM * Number	**Spacing** **Factor**
				**SCMs Content** **kg/m^3^**	**w/cm Ratio**	**Nominal Maximum Aggregate Size (mm)**	**(%)**		**(mm)**
M1	Structural	OPC	362	0.0	0.42	19	6.50	0.20	0.24
M2	Paving	80OPC + 20FA	326	82	0.38	38	5.00	0.10	0.16
M3	Paving	80OPC + 20FA	290	72	0.43	38	6.50	0.36	0.34
M4	Structural	80OPC + 20FA	297	74	0.40	19	6.50	0.39	0.36
M5	Structural	80OPC + 20FA	312	78	0.39	19	6.50	0.10	0.15
M6	Structural	70OPC + 20FA + 10SF	253	109	0.42	19	7.50	0.35	0.15
M7	Structural	70OPC + 20FA + 10SF	208	118	0.40	19	7.50	0.25	0.18
M8	Structural	70OPC + 20FA + 10SF	218	94	0.39	19	7.50	0.37	0.16
**(b)**									
**Mixtures**		**M1**	**M2**	**M3**	**M4**	**M5**	**M6**	**M7**	**M8**
Coarse Aggregates (kg/m^3^)		1100	1070	1020	985	1045	1100	1070	1045
Fine Aggregates (kg/m^3^)		640	685	740	800	596	640	685	596

* SAM = Super Air Meter, OPC: ordinary Portland cement, FA: fly ash, SF: silica fume.

**Table 2:** Chemical composition of cement, silica fume, and fly ash.

Chemical Composition	Cement (% by Mass)	Silica Fume (% by Mass)	Fly Ash-Class F (% by Mass)
CaO	61.18	0.49	1.41
SiO_2_	20.01	95.30	60.56
Al_2_O_3_	4.98	0.20	32.67
Fe_2_O_3_	4.88	0.10	4.44
MgO	1.78	0.27	0.23
SO_3_	2.36	0.24	0.02
Loss on ignition	2.18	1.99	0.21
Total Chloride Content	0.03	-	0.01
Na_2_O	0.20	-	0.02
K_2_O	0.60	-	0.03
Insoluble residue	1.23	-	0.46
Bogue compound composition of cement			
Compound			% (by mass)
C_3_S			49.82
C_2_S			19.78
C_3_A			4.94
C_4_AF			14.84

**Table 3:** Deicing chemical and deicing scaling result.

Mixture	Deicing Chemical
M1	Salt brine
M2	Salt brine
M3	Freeze Guard + bud MgCl_2_
M4	Freeze Guard + bud MgCl_2_
M5	Salt brine
M6	Salt brine
M7	Freeze Guard + bud MgCl_2_
M8	Salt brine
Rating Grade	Rating Description
0	No scaling
1	Very slight scaling (3 mm depth, max, no coarse aggregate visible)
2	Slight to moderate scaling
3	Moderate scaling (some coarse aggregate visible)
4	Moderate to severe scaling
5	Severe scaling (coarse aggregate visible over the entire surface

**Table 4:** Acid soluble Chloride Concentration.

Mixture	Depth (mm)	Acid Soluble Chloride (% by Mass of Cement)	ACI 222R (% by Mass of Cement)- Table 3.1 Threshold
M1	0.0–12.5	0.1240	0.1000
	12.5–25.0	0.1050	
	25.0–37.5	0.0850	
	37.5–62.5	0.0628	
	62.5–75.0	0.0587	
M4	0.0–12.5	0.0754	0.1000
	12.5–25.0	0.0563	
	25.0–37.5	0.0432	
	37.5–62.5	0.0382	
	62.5–75.0	0.0105	
M5	0.0–12.5	0.1109	0.1000
	12.5–25.0	0.1010	
	25.0–37.5	0.0795	
	37.5–62.5	0.0653	
	62.5–75.0	0.0553	
M6	0.0–12.5	0.0533	0.1000
	12.5–25.0	0.0533	
	25.0–37.5	0.0531	
	37.5–62.5	0.0529	
	62.5–75.0	0.0515	
M7	0.0–12.5	0.0534	0.1000
	12.5–25.0	0.0533	
	25.0–37.5	0.0532	
	37.5–62.5	0.0531	
	62.5–75.0	0.0526	
M8	0.0–12.5	0.0530	0.1000
	12.5–25.0	0.0528	
	25.0–37.5	0.0526	
	37.5–62.5	0.0525	
	62.5–75.0	0.0519	

**Table 5:** Summary of TGA and LT-DSC Result.

Mixture	Deicing Chemical	Average CH (g/100 g Paste) *	Reaction Product	Amount of Reaction Product (g/100 g Paste) *
M1	Salt brine	19.54 (1.12)	N-A-S-H	38.65 (4.32)
M2	Salt brine	15.65 (1.06	N-A-S-H	29.65 (4.28)
M3	Freeze Guard + bud MgCl_2_	14.63 (1.23)	M-O-C	28.65 (3.68)
M4	Freeze Guard + bud MgCl_2_	13.65 (1.11)	M-O-C	25.06 (4.63)
M5	Salt brine	13.64 (1.01)	M-O-C	24.96 (3.87)
M6	Salt brine	13.12 (0.86)	M-O-C	14.01 (0.69)
M7	Freeze Guard + bud MgCl_2_	12.98 (0.85)	M-O-C	13.66 (1.01)
M8	Salt brine	11.45 (0.78)	N-A-S-H	12.46 (1.24)

* standard deviation in parenthesis.

**Table 6:** Mass loss and Elastic Modulus retained at 300 F-T cycles.

Mixture ID	Percentage Mass Loss	Percentage Elastic Modulus Retained
M1	0.38	82
M2	0.24	78
M3	0.58	80
M4	0.45	76
M5	0.20	80
M6	0.20	85
M7	0.15	80
M8	0.16	80

**Table 7:** Surface resistivity of the Concrete Mixtures.

Mixture	Average Resistivity (Kilo-Ohms Cm)	Standard Error	Risk Level
M1	17.9	0.4	Moderate risk
M2	73.2	0.5	Low risk
M3	64.6	1.2	Low risk
M4	93.9	5.4	Low risk
M5	109.8	6.9	Negligible risk
M6	145.0	2.5	Negligible risk
M7	163.5	4.8	Negligible risk
M8	178.0	6.5	Negligible risk

**Table 8:** Compressive strength before and after soaking of specimens in deicing chemicals.

Mixture ID	*f*′*_c_*-28 Days (MPa) (Standard Deviation [MPa])	*f*′*_c_*-548 Days (Control) (MPa) (Standard Deviation [MPa])	*f*′*_c_*-548 Days (Soaked) (MPa) (Standard Deviation [MPa])	Percentage Loss (%)
M1	33.58 (1.10)	38.50 (0.14)	34.84 (0.25)	9.50
M2	37.99 (1.65)	39.54 (0.25)	34.20 (0.21)	13.50
M3	35.58 (1.79)	37.68 (0.85)	37.08 (0.15)	1.60
M4	47.57 (0.90)	48.65 (1.25)	37.48 (1.50)	22.95
M5	29.72 (1.03)	32.35 (0.98)	25.78 (0.19)	20.30
M6	52.12 (0.97)	54.32 (1.32)	49.38 (2.65)	9.10
M7	56.64 (0.97)	58.23 (0.85)	50.89 (3.50)	12.60
M8	55.65 (1.62)	58.36 (0.95)	53.40 (3.40)	8.05

**Table 9:** Overview of Alternative Mixtures properties.

Basic Mixture ID	Description	Slump (in)	Air Content (%)	Unit Weight (kg/m^3^)	SAM Number	Type of Deicer	Visual Rating
M1	Original mix (M1_original)	1 1/2	N/A	N/A	N/A	Salt brine	5.0 (Severe)
	Ternary mix (M1_20FA + 10SF)	2	3.25	2290.64	0.43	Salt brine	0 (No scaling)
	40% fly ash (M1_40FA)	N/A	3.5	2287.44	0.54	Salt brine	5.0 (Severe)
M4	Original mix (M4_original)	6 ¾	5.3	2377.14	0.47	Mag Bud Converse	2.0 and ASR
	Plain mix (M4_15FA)	6.5	4.3	2426.79	0.47	Mag Bud Converse	4.0 (severe scaling) and ASR
	Ternary mix (M4_20FA + 10SF)	6 ¾	5.6	2410.77	0.25	Mag Bud Converse	0 (No scaling)
	40% fly ash (M4_40FA)	N/A	2	2324.28	0.4	Mag Bud Converse	1.0 (very slight scaling) and ASR
M8	Plain mix (M8_15FA)	2	5.2	2298.64	0.27	Salt brine	4.0 (Moderate to severe scaling)
	Ternary mix (M8_20FA + 10SF)	1 ¾	5.2	2240.98	0.41	Salt brine	0 (No Scaling)
	40% fly ash (M8_40FA)	6 ¾	4.9	2281.03	0.26	Salt brine	5.0 (Severe)

**Table 10:** An overview of the results of the surface resistivity tests for the different concrete mixtures [16].

District	Basic Mixture ID	Description	Resistivity (Kilo-Ohms-Cm)	Standard Error	Remarks (Chloride Penetrability)
1	(M1)	Original mix (M1_original)	17.9	0.4	Moderate risk
		Ternary mix (M1_ternary)	145.0	2.5	Negligible
		40% fly ash (M1_40FA)	63.0	1.5	Low risk
2	(M4)	Original mix (M4_original)	93.9	5.4	Low risk
		Plain mix (M4_plain)	45.8	4.3	Moderate risk
		Ternary mix (M4_ternary)	163.5	4.8	Negligible
		40% fly ash (M4_40FA)	65.4	2.6	Low risk
6	(M8)	Original mix (M8_original)	109.8	6.9	Negligible
		Plain mix (M8_plain)	58.7	1.5	Low risk
		Ternary mix (M8_ternary)	178	6.5	Negligible
		40% fly ash (M8_40FA)	79	2.7	Low risk

## Data Availability

The original contributions presented in this study are included in the article. Further inquiries can be directed to the corresponding author.
